# Intra-articular hyaluronic acid injection versus oral non-steroidal anti-inflammatory drug for the treatment of knee osteoarthritis: a multi-center, randomized, open-label, non-inferiority trial

**DOI:** 10.1186/ar4446

**Published:** 2014-01-21

**Authors:** Muneaki Ishijima, Toshitaka Nakamura, Katsuji Shimizu, Kunihiko Hayashi, Hiraku Kikuchi, Satoshi Soen, Go Omori, Toshihiko Yamashita, Yuji Uchio, Junji Chiba, Yuki Ideno, Mitsuaki Kubota, Hisashi Kurosawa, Kazuo Kaneko

**Affiliations:** 1 Department of Medicine for Orthopaedics and Motor Organ, Juntendo University Graduate School of Medicine, 2-1-1, Hongo, Bunkyo-ku, Tokyo 113-8421, Japan; 2 Department of Orthopaedic Surgery, University of Occupational and Environmental Health, 1-1, Iseigaoka, Yahatanishi-ku, Kitakyushu 807-8555, Japan; 3 Department of Orthopaedic Surgery, Gifu University, School of Medicine, 1-1, Yanagido, Gifu City 501-1193, Japan; 4 Department of Basic Medical Sciences, School of Health Sciences, Faculty of Medicine, Gunma University, 3-39-22, Showa-machi, Maebashi, Gunma 371-8511, Japan; 5 Department of Orthopaedic Surgery, Sakai Hospital Kinki University Faculty of Medicine, 2-7-1, Harayamadai, Minami-ku, Sakai-city 590-0132, Osaka, Japan; 6 Department of Orthopaedic Surgery and Rheumatology, Nara Hospital Kinki University Faculty of Medicine, 1481-1, Otodacho, Ikoma, Nara 630-0293, Japan; 7 Department of Health and Sports, Niigata University of Health and Welfare, 1398, Shimamicho, Niigata Kita-ku, Niigata 950-3198, Japan; 8 Department of Orthopaedic Surgery, Sapporo Medical University School of Medicine, South-1, West-16, Chuo-ku, Sapporo 060-8543, Japan; 9 Department of Orthopaedics, Shimane University, School of Medicine, 89-1, Enya-cho, Izumo-shi, Shimane 693-8501, Japan; 10 Department of Orthopaedic Surgery, Tokyo Women’s Medical University, Medical Center East, 2-1-10, Nishiogu, Arakawa, Tokyo 116-8567, Japan

## Abstract

**Introduction:**

While many of the commonly used conservative treatments for knee osteoarthritis (OA) have been recognized to be effective, there is still insufficient evidence available. Among the pharmacological treatments for knee OA, oral non-steroidal anti-inflammatory drugs (NSAIDs) act rapidly and are recommended for the management of OA. However, frequent and serious adverse effects of NSAIDs have been recognized. Intra-articular injections of hyaluronic acid (IA-HA) for the treatment of knee OA have been shown to reduce pain and improve joint function. However, there has been no qualified direct comparison study of the efficacy and safety between IA-HA and NSAIDs for patients with knee OA. The aim of this study was to clarify the efficacy and safety of early-phase IA-HA in comparison to those of NSAIDs for patients with knee OA.

**Methods:**

This multicenter, randomized, open-label, parallel-group, non-inferiority comparison study with an oral NSAID involved a total of 200 patients with knee OA. An independent, computer-generated randomization sequence was used to randomly assign patients in a 1:1 ratio to NSAIDs three times per day for five weeks (n = 100) or IA-HA once a week for five weeks (n = 100). The primary endpoint was the percentage change in the patient-oriented outcome measure for knee OA, the Japanese Knee Osteoarthritis Measure (JKOM) score. All patients were questioned regarding any adverse events during treatment. The full analysis set (FAS) was used for analysis. The margin of non-inferiority was 10%.

**Results:**

The analyses of primary endpoint included 98 patients in the IA-HA group and 86 patients in the NSAID group. The difference in the percentage changes of the JKOM score between the two intervention arms (IA-HA; -34.7% (*P*<0.001), NSAID; -32.2% (*P*<0.001)) was -2.5% (95% confidence interval (CI): -14.0 to 9.1), indicating IA-HA was not inferior to NSAID. The frequency of both withdrawal and adverse events in the IA-HA group were significantly lower than those in the NSAID group (*P* = 0.026 and 0.004, respectively).

**Conclusions:**

The early efficacy of IA-HA is suggested to be not inferior to that of NSAIDs, and that the safety of the early phase of IA-HA is superior to that of NSAIDs for patients with knee OA.

**Trial registration:**

UMIN Clinical Trials Registry (UMIN*-*CTR), UMIN000001026.

## Introduction

Osteoarthritis (OA) is an increasingly important public-health problem [1]. The total societal cost of the treatment of OA has been estimated to increase worldwide because of its dramatic growth in morbidity [2]. The current treatment for knee OA consists of conservative treatment, such as exercise, physical therapy, pharmacological agents and, in some cases, surgical treatment [3,4]. While many of the commonly used conservative treatments have been recognized to be effective [5], there is still insufficient evidence available.

Among the pharmacological treatments for knee OA, oral non-steroidal anti-inflammatory drugs (NSAIDs) act rapidly and are recommended for the management of OA, although frequent and serious adverse effects of NSAIDs have been recognized [5]. Hyaluronic acid (HA) is a natural constituent of joint fluid. Intra-articular injections of HA (IA-HA) for the treatment of knee OA have been shown to reduce the pain and improve joint function [5-7]. Although IA-HA is also recommended, it acts relatively slowly and there was considerable heterogeneity in the outcomes between trials [8-11]. In addition, there has been no qualified direct comparison study of efficacy and safety between IA-HA and NSAIDs for patients with knee OA.

The aim of this multicenter, randomized, parallel-group, open-label, non-inferiority trial was to compare the early efficacy and safety of IA-HA and NSAIDs in patients with knee OA.

## Methods

### Study design and participants

The trial was planned by the Cartilage Metabolism Research Group, consisting mainly of Japanese orthopedists, to clarify the early efficacy and safety of IA-HA (high molecular weight 2,700 kDa HA, Chugai Pharmaceutical Co. Ltd., Tokyo, Japan) in comparison to an NSAID (loxoprofen sodium, Daiichi Sankyo Pharmaceuticals Co. Ltd., Tokyo, Japan), in a multicenter, randomized, open-label, parallel-group, non-inferiority trial. The protocol was reviewed and approved by the ethics committee of Juntendo University, Tokyo, Japan, and was also reviewed by the institutional review board of each participating institution. This study was undertaken at 20 hospitals throughout Japan between February, 2008 and December, 2010 (see Acknowledgements), in accordance with the Declaration of Helsinki, and with the Ethical Guidelines for Clinical Studies of the Japanese Ministry of Health, Labor, and Welfare. This trial was registered at UMIN-CTR [12], UMIN000001026.

### Subjects

All patients provided written informed consent before enrollment in this trial. The inclusion criteria for the present study included (1) subjects who were able to walk with painful knee OA and fulfilled the criteria for knee OA of the medial femorotibial joint as defined by the American College of Rheumatology (ACR) [13], (2) the age of the subjects ranged from 50 to 80, (3) female subjects were required to be postmenopausal, and (4) all subjects had radiographic knee OA with Kellgren-Lawrence (K/L) grade 1 to 3 [14] evaluated by the weight-bearing anteroposterior X-rays of the tibiofemoral joint using the bilateral standing extended view.

The exclusion criteria included (1) patients who had received either an oral, topical or intra-articular steroid during the four weeks before the study, (2) patients who had received IA-HA within four weeks before the study, (3) patients who had received either an oral, topical or suppository NSAID within two weeks before the study, (4) patients who had secondary knee OA, (5) patients with patellofemoral OA with a K/L grade of 3 or higher, (6) patients with severe OA (K/L grade 3 or higher) in a location other than the knee joint, (7) patients with rheumatoid arthritis, (8) patients who had received joint replacement surgery in either knee or/and a hip, (9) patients who had allergies to either HA or NSAIDs, (10) patients who had either hematological, cardiac, hepatic or renal disorders, (11) patients who had experienced an asthma attack induced by NSAIDs, and (12) patients whom the physician recognized as not suitable for enrollment in the study for other reasons.

### Randomization and masking

A centralized, computer-generated randomization was conducted to randomly assign patients in a 1:1 ratio to the IA-HA or NSAID groups. Investigators were masked to assignment before, but not after, randomization. The website for patient registration and randomization was prepared and controlled by the coordinating data center (Gunma University, Maebashi, Japan). The blocked randomization was stratified by the participating medical center and the K/L grade of knee OA.

### Treatment procedures

A total of 200 patients with symptomatic knee OA were registered from 20 hospitals and randomized for treatment with the NSAID or IA-HA, as described above. For patients treated with the NSAID, they received three daily 60 mg NSAID tablets (total 180 mg)/day, one after each meal, for five weeks. Additional use of gastro-protective drugs, such as a proton pump inhibitor (PPI), in combination with the NSAID was allowed for those in the NSAID group. For patients treated with IA-HA, an intra-articular injection of high-molecular-weight 2,700 kDa HA (25 mg) was administered into the affected joint five times, at weekly intervals in the morning. Concomitant use of other drugs for the treatment of OA and drugs that affect bone and cartilage metabolism were not allowed during the trial.

### Outcome measures for the assessment of efficacy and safety

The patients were evaluated for their (1) baseline characteristics, (2) radiographic analysis of the knee, (3) compliance with the treatment, (4) clinical manifestations, and (5) safety.

### Evaluation of the response to treatment (efficacy)

Pain was evaluated by a visual analog scale (VAS, 0 to 100). The clinical manifestations were evaluated by the Japanese Knee Osteoarthritis Measure (JKOM) score [15]. The JKOM is a patient-based, self-answered evaluation score that includes four subcategories: pain and stiffness (0 to 32), activities of daily living (0 to 40), social activities (0 to 20), and general health conditions (0 to 8) with 100 points as the maximum score. The JKOM score is higher in patients with more pain and physical disability, and this evaluation modality is considered to have sufficient reliability and validity for studies of the clinical outcomes of Japanese subjects with knee OA [15]. The measure has also been shown to have reliability and validity by means of statistical evaluation and comparison with other health-related scales, such as the Western Ontario and McMaster Universities Arthritis Index (WOMAC) and the Medical Outcomes Study 36-Item Short-Form Health Survey (SF-36) [15].

The primary endpoint was to compare the percentage change from baseline in the JKOM score at five weeks. The secondary endpoint was to compare the percentage change from baseline in the pain VAS score.

The definition of a response to treatment was made following the criteria defined by the Outcome Measures in Rheumatology Clinical Trials (OMERACT) and Osteoarthritis Research Society International (OARSI) [16]. This measure consists of both absolute and relative changes in scales, including both pain and function, to evaluate the affected knee. Relative change means the percentage of change during the study (final minus baseline over baseline × 100), whereas absolute change indicates the absolute change during the study (final minus baseline on an interval scale of 0 to 100). Before assessing patients based on this scale, we partly modified it for this study by using the JKOM score, as already reported [17,18]. The response was defined as relief of joint pain or improvement in function (at least 50% reduction of the score) and a decrease of at least 20 mm on the VAS, or clinical improvement meeting at least two of the following three conditions: a decrease in joint pain of at least 20% and at least 10 mm on the VAS; an improvement in function of at least 20% and a decrease of at least 4 points from a total 40 points (equal to an absolute change of 10%) on the JKOM functional subcategory scale; and a decrease in the patient’s global assessment score by at least 20% and at least 10 points from a total of 100 on the total JKOM scale.

### Assessment of adverse events induced according to the treatment modality (safety)

Safety was monitored by recording all adverse events, evaluating the laboratory data and assessing vital signs. This was performed for all participants in both groups at each weekly visit.

### Statistical analysis

#### Sample size determination

The trial was designed to establish whether the symptom-modified effect of IA-HA was non-inferior to that of NSAID (Δ10%). The sample size of this non-inferiority trial was calculated to require a total of 194 patients (97 per treatment group) based on the results of our pilot study with a 5% dropout rate, 10% non-inferiority margin, 27% standard deviation (SD), 5% one-sided alpha level, and power = 0.8 (pilot study: Toshitaka Nakamura, unpublished data, 2007). The 10% margin was set as the smallest value that would be clinically important, assuming a reduction of 30% in the mean percentage change of JKOM score in patients with both IA-HA and NSAID treatment and a reduction of 10% those receiving a placebo treatment.

### Data analysis

The primary statistical analyses of efficacy and safety were performed on the full analysis set (FAS), which included all patients treated at least once. For the primary endpoint of the study, a two-sided 95% confidence interval (CI) for the group difference ‘test treatment minus reference treatment’ was calculated for the percentage change from baseline in the JKOM score as non-inferiority analysis. The non-inferiority of the test treatment was confirmed if the upper limit of the CI was ≤ margin of non-inferiority delta (10%). For the secondary endpoint, the group difference and its 95% CI was calculated for the percentage change from baseline in the VAS pain score.

Quantitative variables were described using the mean, standard deviation and range. The efficacy of treatment was examined by a paired *t* test for both JKOM score and pain VAS score. A multiple logistic regression analysis was used to estimate odds ratios and their 95% CIs between the IA-HA and NSAID treatments in models adjusted for age, K/L grade, body mass index (BMI) and the participating medical centers.

All analyses were performed using the SAS System Release 9.1 software program (SAS Institute, Cary, NC, USA). The registration number of this trial is UMIN000001026, and information on the trial can be found online at [12].

## Results

### Patient baseline characteristics

**Figure 1:**
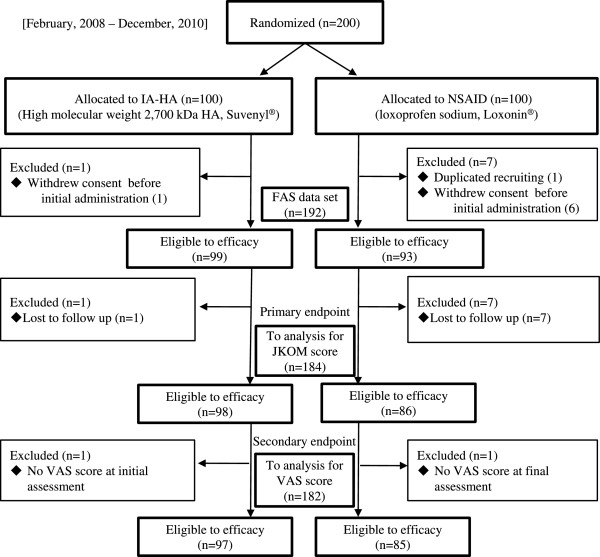
**A flow chart of the study.** FAS, full analysis set; HA, hyaluronic acid; IA-HA, intra-articular injections of HA; ITT, intention-to-treat; JKOM, Japanese Knee Osteoarthritis Measure (JKOM); NSAID, non-steroidal anti-inflammatory drug; VAS; visual analog scale.

A flow chart of this trial is shown in Figure 1. When 200 patients were enrolled, half (100) of the patients were randomly allocated into the NSAID group, and the other half allocated into the IA-HA group. Two patients in the IA-HA group and 14 patients in the NSAID group were excluded; therefore, the remaining 184 patients were included in the analyses of the primary endpoint.

The baseline patient characteristics are shown in Table 1. No significant statistical differences between the baseline characteristics of both groups were found.

**Table 1:** Baseline characteristics of the patients in the study

		**IA-HA**	**NSAID**
		**(n = 99)**	**(n = 93)**
Age (y)	Mean (SD)	68.2 (7.1)	68.5 (7.0)
Gender	Male	27	22
	Female	72	71
BMI	Mean (SD)	23.8 (3.4)	24.4 (3.6)
K/L grade	1	16	15
	2	48	50
	3	35	28
JKOM score (Min:0 - Max:100)	Mean (SD)	33.8 (15.8)	31.6 (14.1)
Pain VAS (Min:0 - Max:100)	Mean (SD)	60.3 (22.4)	55.1 (21.9)

IA, intra-articular; HA, hyaluronic acid; NSAID, non-steroidal anti-inflammatory drug; BMI, body mass index; K/L, Kellgren-Laurence grade; JKOM, Japanese Knee Osteoarthritis Measure; VAS, visual analog scale.

### Efficacy analyses (primary and secondary endpoints)

For the primary endpoint analysis, the JKOM score of the patients in both the NSAID group and in the IA-HA group was significantly reduced by the treatment (*P*<0.001, Table 2), and the percentage change from baseline in the JKOM score for the two groups was -32.2% and -34.7%, respectively. The difference in the percentage changes of the JKOM score between the two intervention arms (primary endpoint) was -2.5% (95% CI: -14.0 to 9.1%).

**Table 2:** Results of the primary endpoint of the study

	**JKOM score**				**% change of JKOM score**		
		**Mean**	**SD**	***P*** **(post vs. pre)**	**Mean (%)**	**SD**	**Difference (%) [IA-HA - NSAID] (95% CI)**
IA-HA (n = 98)	Pre-treatment	33.8	15.9	<0.001	-34.7	39.6	-2.5 (-14.0 to 9.1)
	Post-treatment	21.5	14.6				
NSAID (n = 86)	Pre-treatment	32.0	14.0	<0.001	-32.2	39.8	
	Post-treatment	22.0	15.5				

The effect of the treatment of either IA-HA or NSAID for the patients with knee OA evaluated by JKOM score (left) and percentage (%) change of JKOM score (right). A *P* value less than 0.05 was considered to be significant. JKOM, Japanese Knee Osteoarthritis Measure; IA, intra-articular; HA. hyaluronic acid; NSAID, non-steroidal anti-inflammatory drug.

In a multiple regression analysis performed taking into consideration the factors considered to stratify the study design, the difference in the primary endpoint between the two intervention arms was also less than 10% (data not shown). These results demonstrate that the IA-HA treatment was non-inferior to the NSAID treatment for the percentage reduction in the clinical symptoms evaluated by the JKOM.

For the secondary endpoint analysis, the pain VAS score of the patients in the NSAID group was significantly reduced by the treatment (*P*<0.001, Table 3). The percentage change from baseline in the VAS score in the NSAID group was -36.0%. The pain VAS in the IA-HA group was also significantly reduced by the treatment, with a percentage change from baseline in the VAS score of -41.2% (*P*<0.001). The difference in the percentage changes in the pain VAS score between the two intervention arms (secondary endpoint) was -5.2% (95% CI: -23.8 to 13.4%).

**Table 3:** Results of the secondary endpoint of the study

	**Pain VAS**				**% change of VAS score**		
		**Mean**	**SD**	***P*** **(post vs. pre)**	**Mean (%)**	**SD**	**Difference (%) [IA-HA - NSAID] (95% CI)**
IA-HA (n = 97)	Pre-treatment	60.1	22.4	<0.001	-41.2	52.7	-5.2 (-23.8 to 13.4)
	Post-treatment	31.8	24.1				
NSAID (n = 85)	Pre-treatment	55.5	21.8	<0.001	-36.0	73.8	
	Post-treatment	31.9	23.9				

The effect of the treatment of either IA-HA or NSAID for the patients with knee OA evaluated by pain VAS score (left) and percentage (%) change of VAS score (right). A *P* value less than 0.05 was considered to be significant. VAS, visual analog scale; IA, intra-articular; HA, hyaluronic acid; NSAID, non-steroidal anti-inflammatory drug.

### Subanalyses

When the patients were divided into two groups (responders or non-responders) by the OMERACT-OARSI response criteria [16], 69.7% (69/99) of the patients in IA-HA group were classified as ‘responders’, while 62.4% responders were found (58/93) in the NSAID group. Again, there were no significant differences in the frequency of ‘responders’ between these two groups (*P* = 0.283).

A multiple logistic regression analysis, which was adjusted for age, K/L grade, BMI and the participating medical centers, confirmed the lack of significant differences in the odds ratio of responders between those who received IA-HA treatment and those who received NSAID treatment (odds ratios: 1.47 (95% CI: 0.761 to 2.83)).

We further investigated whether IA-HA is broadly effective from very early (K/L grade of 1) to moderate stages of knee OA (K/L grade of 3) (Table 4). Both IA-HA and NSAID groups significantly reduced the patient-oriented outcome measure evaluated by the JKOM score in the patients with both a K/L grade of 2 and 3. In patients with a K/L grade of 1, IA-HA treatment also reduced the JKOM score, but this reduction was not significant (*P* = 0.058). On the other hand, NSAID treatment of this group significantly reduced the JKOM score (*P* = 0.001).

**Table 4:** JKOM score and percentage change of JKOM score by K/L grade subgroup

	**JKOM score**				**% change of JKOM score**		
		**Mean**	**SD**	***P*** **(post vs. pre)**	**Mean (%)**	**SD**	**Difference (%) [IA-HA - NSAID] (95% CI)**
K/L grade 1							
IA-HA (n = 15)	Pre-treatment	24.8	13.0	0.058	-9.3	78.0	25.7 (-19.3 to 70.7)
	Post-treatment	18.7	12.6				
NSAID (n = 14)	Pre-treatment	35.9	15.4	0.001	-34.9	26.0	
	Post-treatment	23.4	15.7				
K/L grade 2							
IA-HA (n = 48)	Pre-treatment	33.1	14.7	<0.001	-43.8	27.1	-9.2 (-24.4 to 6.0)
	Post-treatment	18.8	12.6				
NSAID (n = 45)	Pre-treatment	30.8	13.9	<0.001	-34.6	44.9	
	Post-treatment	20.4	14.5				
K/L grade 3							
IA-HA (n = 35)	Pre-treatment	38.6	17.1	<0.001	-33.1	23.6	-6.2 (-21.6 to 9.3)
	Post-treatment	26.4	16.9				
NSAID (n = 27)	Pre-treatment	31.9	13.6	0.003	-26.9	37.0	
	Post-treatment	23.7	17.3				

A *P* value less than 0.05 was considered to be significant. JKOM, Japanese Knee Osteoarthritis Measure; IA, intra-articular; HA, hyaluronic acid; NSAID, non-steroidal anti-inflammatory drug; K/L, Kellgren-Laurence grade.

### Safety analyses

During the five weeks of examination, nine of ninety-nine patients (9.1%) in the IA-HA group were withdrawn from the study (one patient’s symptoms improved and eight patients were lost to follow-up). Nineteen of ninety-three patients (20.4%) of NSAID group were withdrawn from the study (five patients experienced side effects, four withdrew consent, two patient’s symptoms improved, and eight were lost to follow-up). The frequency of the withdrawal rate in the IA-HA group was significantly lower than that in the NSAID group (*P* = 0.026, Table 5).

**Table 5:** Withdrawal and harmful events during the study

**Withdrawn**	**Completed**	**Withdrawn**	**Frequency (%)**	***P***
IA-HA (n = 99)	90	9	9.1	0.026
NSAID (n = 93)	74	19	20.4	
**Harmful events**	**Not occurred**	**Occurred**	**Frequency (%)**	***P***
IA-HA (n = 99)	98	1	1.0	0.004
NSAID (n = 93)	83	10	10.8	

IA, intra-articular; HA, hyaluronic acid; NSAID, non-steroidal anti-inflammatory drug.

Serious adverse events, including gastrointestinal (GI) hospitalization, were not observed in both groups during this study. As one patient complained of stiffness in the affected knee after injection, the frequency of adverse events in patients treated with the IA-HA was 1.0%. Ten (symptom related to GI tract disorder, seven; drug allergy, three) of ninety-three patients (10.8%) exhibited adverse events in those treated with the NSAID. The frequency of adverse events in the IA-HA group was significantly lower than that of those in NSAID group (*P* = 0.004, Table 5).

## Discussion

This short-term trial clearly demonstrated that both the IA-HA at weekly intervals and daily oral NSAID over five weeks significantly improved both the clinical symptoms evaluated by the patient-oriented outcome measure and the pain severity evaluated by a VAS. No significant differences in the symptom-modifying effects were observed during this short period. In addition, the safety of the early phase of IA-HA treatment was superior to that of the NSAID in the patients with knee OA.

HA is a large glycosaminoglycan composed of repeating disaccharides of glucuronic acid and N-acetyl glucosamine that is naturally present in synovial fluid. Several protective properties of HA have been reported including shock absorption, traumatic energy dissipation, protective coating of the articular cartilage surface, and lubrication [19]. Numerous clinical trials, meta-analyses and systematic reviews have indicated its clinical efficacy for knee OA [5,9,10,20]. Based on these previous findings, the OARSI recommendations that were revised in 2010 summarized the effect size (ES) of IA-HA at 0.60 (95% CI; 0.37, 0.83). However, as the ES declined to 0.22 (95% CI; -0.11, 0.54) when only the high-quality trials were selected [5], controversy remains regarding the efficacy of HA in treating knee OA [8]. A recent meta-analysis concluded that the pain reduction by IA-HA is observed later than that of intra-articular corticosteroids [9]. In addition, the effects of IA-HA for knee OA pain continued over six months post-intervention [10]. However, few studies have been conducted to clarify the early effects and safety of IA-HA in comparison to those of NSAIDs. The results of this study clearly indicated that the early efficacy of IA-HA was not inferior in comparison to that of the NSAID.

A number of HA products with a variety of the molecular weights, ranging from approximately 600 to 6,000 kDa, have been developed as IA-HA for the treatment of OA [8]. The considerable heterogeneity of outcomes between trials may be due in part to differences in HA products [5]. High-molecular-weight HA (>6,000 kDa) is suggested to have greater effects in comparison to lower-molecular-weight HA [8]. On the other hand, the intra-articular injection of high-molecular-weight HA (>6,000 kDa) showed a greater frequency of adverse events, such as pain flares, and hot and swollen knees, which typically occurred 24 to 72 hours after injection [21]. There were no cases of painful, hot or swollen knees during the study.

The molecular mechanisms underlying the efficacy of IA-HA for OA remain unclear. OA is frequently associated with the signs and symptoms of inflammation, including joint pain, swelling and stiffness leading to significant functional impairment and disability [2]. Synovitis plays an important role in inducing the pain, swelling and stiffness in OA [22], and the severity of synovitis is well correlated with the JKOM score of the patients with knee OA [23]. It has recently been reported that HA inhibits the activities of matrix metalloproteinases and aggrecanases which are, at least in part, involved in OA cartilage degradation as a result of their induction by proinflammatory cytokines, such as interleukin (IL)-1 [19,24-26]. Therefore, HA is speculated to modify the structural damage of joints and the rate of OA progression in addition to the symptom-modifying effect [27], although further studies are required.

In this trial, the early efficacy of IA-HA was compared with that of NSAID for the treatment of knee OA. NSAIDs have also been proven to be an effective conservative treatment for knee OA [5]. However, a high incidence of serious GI tract adverse events associated with the use of oral NSAIDs was also demonstrated in a population-based cohort study of older patients [28]. In addition, the hospitalization due to GI tract side effects in patients receiving non-selective NSAIDs was twice as high as that in those given the cyclooxygenase (Cox)-2 selective agent, celecoxib, or a non-selective NSAID together with a PPI [28]. Although a PPI was not routinely used in addition to the NSAID (loxoprofen sodium) in this study, no serious GI events were noted.

Since chronic kidney disease (CKD), which is similar to knee OA, is also a prevalent disease especially in older populations, knee OA patients with CKD may have a different risk profile and treatment response than knee OA patients without CKD. However, as patients with renal disorders were excluded in the present study, as described in the Methods section, whether the presence of CKD has any effect on the efficacy and safety of either the IA-HA or NASIDs remains unclear.

The efficacy of IA-HA for knee OA has been debated for over a decade. Although it has been systemically evaluated in meta-analyses, most previous studies have focused on comparing the findings with either placebo or intra-articular corticosteroids [9,10,29]. No previous studies have undertaken a meta-analysis with NSAIDs, which is one of the most efficacious and widely used treatments for knee OA [5]. The present study clearly shows that IA-HA is as effective as continuous NSAID use at five weeks of treatment, and, in addition, it showed a more favorable safety profile of IA-HA over NSAIDs for knee OA. The present study suggests that future randomized trials should thus be carried out with a longer duration of follow-up and larger samples, in order to identify optimal knee OA treatment alternatives. Furthermore, it would also be interesting to evaluate whether any synergistic effect of these two combined treatments exists when they are combined.

The current study does have some limitations. First, this investigation was an open-label randomized trial and not a double-blind controlled trial. Therefore, the design may have introduced certain bias into the results. Second, the trial’s size was calculated to have sufficient power to exclude a 10% between-group percentage change of JKOM score, which can be debated. This margin was supported by our pilot study, as described previously. Third, in subgroup analysis for the patients with a K/L grade of 1, IA-HA treatment reduced the JKOM score. However, this reduction was not statistically significant (*P* = 0.058). Although the reason for this is unclear, the interpretation of the result was limited by the small number of patients (n = 15) and, therefore, it may be one of the limitations. Even though some subjects had a K/L grade of 1, some have an increased risk for rapid progression of the disease [30]. Unfortunately, we cannot predict radiographically who is at risk for progression [4].

## Conclusions

The early efficacy of IA-HA is suggested to be not inferior to that of a NSAID, and the safety of the early phase of IA-HA is superior to that of a NSAID for patients with knee OA.

## Abbreviations

ACR: American College of Rheumatology; BMI: body mass index; CKD: chronic kidney disease; FAS: full analysis set; GI: gastrointestinal; HA: hyaluronic acid; IA-HA: intra-articular injections of HA; JKOM: Japanese Knee Osteoarthritis Measure; K/L: Kellgren-Lawrence; NSAIDs: non-steroidal anti-inflammatory drugs; OA: osteoarthritis; OARSI: Osteoarthritis Research Society International; OMERACT: Outcome Measures in Rheumatology Clinical Trials; PPI: proton pump inhibitor; SF-36: Medical Outcomes Study 36-Item Short-Form Health Survey; VAS: visual analog scale; WOMAC: Western Ontario and McMaster Universities Arthritis Index.

## Competing interests

The authors declare that they have no competing interests.

## Authors’ contributions

As principal investigators of this study, all authors of this study had full access to all data, and take responsibility for their integrity and the accuracy of their analysis. TN, KS, KH, HKu and KK participated in the study design. HKu and KK supervised the study. MI, TN, KS, HKi, SS, GO, TY, YU, JC, MK and HKu collected the data. MI, YI and KH analyzed the data. YI and KH provided statistical expertise. MI and KH drafted the manuscript, and the manuscript was revised for content by MI, TN, KS, KH, HKi, SS, GO, TY, YU, JC, YI, MK, HKu and KK. All authors read and approved the final manuscript.
